# Prevention of Pulmonary Tuberculosis

**Published:** 1905-01

**Authors:** 


**A Monthly Review of Medicine and Surgery.**

*EDITOR:*

WILLIAM WARREN POTTER, M. D.

All communications, whether of a literary or business nature, books for review and exchanges, should be addressed to the editor: 284 Franklin St., Buffalo, N. Y.

Vol. XLIV.—LX. JANUARY, 1905. No. 6

### Prevention of Pulmonary Tuberculosis.

ON FORMER occasions, when opportunity presented, we have discussed in these columns the tuberculosis problem in its various phases and from several viewpoints. If we return to it again it is because the subject is an important one and, moreover, because it is yet far from solution. It is quite true that progress has been made in a number of directions during the last ten years, but so much remains to be done, not only with reference to the cure of tubercular disease but, even more importantly, concerning its prevention, that the work looks still like a most formidable undertaking.

In spite, however, of discouragements that manifestly seem great, the medical profession must not look backward, but continue to put up its strongest and most intelligent fight against such a subtle and destructive enemy as tuberculosis. Some of these attacks must be made in one direction and some in another, but all must have one purpose in view as a foundation principle—namely, the education of every intelligent person unfamiliar with the prevention of infectious disease. To teach the habit of personal cleanliness should become the cheerful duty of every physician; to teach the practice of municipal cleanliness should be made part of the legal duty of every public health official, and the law relating to this point should be lived up to—even enforced if need be—as to both letter and spirit.

The question of enforcing municipal cleanliness is, perhaps, the most difficult of all measures relating to the whole problem. The tendency of the rural population to congregate in cities is ever increasing; and as the cities grow in size, becoming constantly more and more congested, the difficulties that stand in the way of enforcing sanitary laws are enhanced in a four fold degree. A moment's reflection will make the reason for this plain. In rural districts and smaller villages whence much of the city population is derived, there is little or no attention paid to minor questions of cleanliness. For example, in the country almost every lad acquires the spitting habit which, in itself, may do little harm in the environment of the farmhouse and village blacksmith shop; but when these young persons having removed to the city continue to practise the indecent habit, the question becomes a serious one and the utmost vigilance and stringency are required to overcome it.

In the propagation of tuberculosis there is no more active agent than the person given over to the spitting habit. The reasons for this have been stated over and again, hence need not be repeated here. It is the habit, itself, that we are dealing with in particular at this time. It is more than likely that if this vicious and vile habit could be suppressed in toto, the deaths from tuberculosis would decrease 50 per cent. within ten years. Think, for a moment, what that would mean in the city of Buffalo alone! Think, again, what it would mean in the state of New York!

The reputation which Buffalo bears for cleanliness has been acquired through a general attention to the grosser details, such as street sweeping and frequent removal of garbage, but as yet our ordinances are silent on the subject of spitting on the streets. It is true that in street cars and public places the practice would be prohibited if the ordinances were enforced; and while this makes for something in the right direction, it has not one jot of the importance that suppression of street spitting would entail.

We are prompted to these remarks by a close observation of the sidewalks in the principal streets of Buffalo, extending over a period of more than a year. Let anyone who feels inclined to investigate this subject take a walk along the shopping district of Main street any afternoon when there is no snow,—and this means particularly in the spring, summer and autumn seasons,—and he will be astonished at the spectacle. Men in almost countless numbers are bespitting the streets, and women are wiping it over with their skirts, while the sun and wind are licking it up and converting it into atmospheric particles most convenient for respiration and which, peradventure, in not a few instances, are most fatal in their effects.

It may be stated as a general proposition that no well bred gentleman will spit upon the sidewalk; and yet the writer, during his long period of observation, has rarely seen a pedestrian go to the curb to relieve his salivary reservoir. In a great many cities smaller than Buffalo, ordinances impose a fine for spitting on the sidewalk, and it is high time that something were done in Buffalo to suppress this vile and dangerous practice. It is an abomination against the laws of common decency, and it is a suicidal infraction against what should be a fundamental law of municipal sanitation. Members of the municipal league, physicians and good citizens in general ought to be prevailed upon to unite in an effort to put an end to this tragic violation of the commonest principles of decency and health. Until it is done other measures looking to the prevention of the spread of tuberculosis will fail of their purpose in a very large degree, if they are not rendered entirely futile.

---

Professor Robert Koch, of Berlin, started for South Africa December 17, 1904, to engage in further scientific investigation of rinderpest and other animal diseases. His last trip, devoted to the discovery of remedies for these diseases, was in behalf of the British government. In the course of the professor's investigations some theoretical and scientific problems arose which he did not have time to solve. The present trip will be devoted to the study of these problems.

N. B.—Professor Koch has nothing to do with the so-called koch lung cure deceptions, one of which lately went out of commission in Buffalo.

---

Messrs. Fairchild Brothers & Foster, of New York, in appreciation of the friendly relations which have existed for many years between them and the pharmacists of the United Kingdom, have established the Fairchild scholarship of the value of fifty pounds annually and various consolation prizes of five pounds each, to be awarded on competitive examination to candidates entering in England, Ireland, Scotland, and Wales, who are registered students of pharmacy. The details governing the awards will be supplied by the founders on application, or by Mr. A. E. Holden, Bath House, 57-60 Holborn Viaduct, London, E. C. This is a manifestation of wise liberality on the part of this celebrated house and cannot fail to be appreciated by those whom it is intended to benefit.

---

As if this region of the state were not already amply supplied, not to say infested, with an adequate number of medicinal fakirs, such as "lung curers," Christian science fanatics, and advertising legerdemainists, a new healer is announced as having appeared at Dunkirk. This is the way, according to a "special" to the *Buffalo Express*, in which he made his advent:

* * * who boasts long hair and a longer beard, blew into town today with a game to work healing under the name of Schlatter.

He has a good press agent, who claims the man had been offered $5,000,000 by a famous rich woman to marry her. He refused on the ground that the rich cannot enter heaven. Another rich woman bid $1,000,000 for his hand, but he could not consider that offer in view of the stand he had taken. The stranger has hired a hall and proposes to begin on Sunday the healing of the sick free of charge.

We quite approve this marrying scheme, and suggest it to some of the Buffalo "deceivers" who may be running short of "scare head" items.

---

The conviction of Anton J. Weichers, the "boy wonder" (aged forty years) which was obtained in the Supreme Court of Buffalo nearly two years ago has remained in abeyance pending a decision by the higher courts. The conviction, it will be remembered, was for conspiracy to defraud, in pretending to cure disease without the slightest qualifications as required by law. The Court of Appeals has affirmed the conviction, and the remainder of the sentence must be served out in the penitentiary.

---

Pure milk is, next to pure water, the most important desideratum in a large city. We note with interest that the Medical Society of the County of New York, the Dairy Division of the United States Department of Agriculture, the Milk Commission and the Board of Health of New York, have combined in a movement to provide a guaranteed milk supply.

This milk is not to be sold in bulk, but only in sealed bottles. Every bottle will have a cap on which will be stamped: "Certified by the Commission of the Medical Society of the County of New York." These caps will be issued under the authority of the medical society direct to the producers or dealers. By this means any adulteration or lack of quality in the milk can be traced at once to the producer.

It would be well if a similar system were adopted in relation to the milk supply of Buffalo. We commend the subject as worthy the attention of the Medical Society of the County of Erie.

---

Attention is invited to the address to the medical profession of the chairman of the legislative committee of the Medical Society of the State of New York, Dr. Frank Van Fleet, on the optometry campaign which the mechanical opticians are getting ready to wage with the next legislature, published under Topics of Public Interest. It is only necessary to be forewarned to be forearmed.

